# Elevated quinolizidine alkaloid content in grains of sweet narrow‐leaved lupins when intercropped with oats

**DOI:** 10.1002/jsfa.70396

**Published:** 2026-02-11

**Authors:** Yannik Schlup, Patrick PJ Mulder, Sylvia Kalli, Monique de Nijs, Johan Six, Susanne Vogelgsang

**Affiliations:** ^1^ Research group Extension Arable Crops, Competence Division Plants and Plant Products Zurich Switzerland; ^2^ Sustainable Agroecosystems, Department of Environmental Systems Science ETH Zurich Zurich Switzerland; ^3^ Wageningen Food Safety Research Wageningen University and Research Wageningen Netherlands

**Keywords:** quinolizidine alkaloid, narrow‐leaved lupin, *Lupinus angustifolius*, mixed cropping, intercropping, food safety

## Abstract

**BACKGROUND:**

Narrow‐leaved lupins (NLL, *Lupinus angustifolius* L.) is recognized as a climate‐resilient protein crop but its use in food and feed is frequently limited by toxic quinolizidine alkaloids (QAs). The effect of intercropping with spring oat (*Avena sativa* L.) on grain QA content has not yet been quantified.

**RESULTS:**

In a 2‐year field experiment, three NLL varieties (*Lunabor*, *Probor*, and *Jowisz*), grown as pure stands and in nine mixtures with the oat varieties *Bison*, *Lion*, and *Troll* were compared. Mixed cropping increased total grain QAs by between 16% and 46% relative to the respective pure stands. Absolute increases reached +168 mg kg^−1^ in *Lunabor* and +128 mg kg^−1^ in *Probor*, whereas *Jowisz* increased by only +76 mg kg^−1^. Among the mixtures, *Jowisz*–*Bison* exhibited the smallest increase (16%) and the lowest final QA content, whereas *Lunabor*–*Troll* showed the highest content. In mixed stands, both *Lunabor* and *Probor* exceeded the 500 mg kg^−1^ threshold, whereas *Jowisz* remained below this threshold. Profiles of the seven major QAs remained constant, with the exception of the 13‐hydroxylupanine to lupanine ratio, which increased in the mixture. Year effects were not observed.

**Conclusion:**

Intercropping NLL with oat elevates the grain QA content to levels of toxicological relevance. The extent is variety‐interaction dependent, presumably due to oat allelopathy. The evidence points to an indirect stress mechanism: allelopathic cues from the oat crop place NLLs under physiological stress, which in turn stimulates NLL to accumulate additional QAs in the grain. Additional mixed cropping experiments and breeding against QA accumulation in NLL grains should be pursued to understand and alleviate this issue. © 2026 The Author(s). *Journal of the Science of Food and Agriculture* published by John Wiley & Sons Ltd on behalf of Society of Chemical Industry.

## INTRODUCTION

Climate change and increased demands for sustainably produced protein have generated interest in narrow‐leaved lupin (NLL) (*Lupinus angustifolius*) – a valuable legume with the potential to increase sustainable and local plant protein production.^1^, ^2^, ^3^ Owing to frost tolerance down to −10 °C and high drought tolerance, NLL is well suited to cultivation under climate change.^4^, ^5^, ^6^ Its extensive and specialized root system can fix up to 300 kg ha^−1^ of atmospheric nitrogen (N) and mobilize 30 kg ha^−1^ insoluble phosphorus (P), effectively reducing input requirements and the associated carbon footprint during cultivation.^7^, ^8^, ^9^ In contrast with white lupins (*Lupinus albus* L.), NLL is tolerant to anthracnose, a fungal disease caused by *Colletotrichum lupini*.^4^ Anthracnose can lead to complete yield loss, making disease tolerance an essential trait.^10^, ^11^ Collectively, these attributes make NLL a strong pulse crop candidate for temperate zones under climate change and explain why NLL is the most widely cultivated lupin species worldwide.^4^, ^12^

Narrow‐leaved lupins' nutritional profiles exhibit some of the highest protein content in the plant kingdom, comparable with the protein value of soybean.^1^, ^3^ The protein and fat profiles of NLLs, along with their dietary fiber and mineral content make them exceptionally well suited for human nutrition, surpassing the nutritional quality of other legume crops.^13^, ^14^, ^15^ Despite their allergenic potential, the long history of lupin cultivation is thus not surprising.^4^ However, the accumulation of quinolizidine alkaloids (QAs), toxic secondary metabolites that protect lupins against pests, microbes, and herbivores, limits their use in food and feed.^16^, ^17^, ^18^ Sparteine and other QAs are highly neurotoxic and cardiotoxic for humans. Using a margin‐of‐exposure approach, the European Food Safety Authority (EFSA) panel established a lowest single oral effective dose of 0.16 mg kg^−1^ body weight as reference point for acute exposure for humans.^19^, ^20^, ^21^, ^22^

The QA content and composition are primarily defined by the genetic background of the NLL variety.^16^, ^17^, ^23^ Narrow‐leaved lupin grains naturally contain 1% to 8% QAs of grain dry mass. The discovery of sweet mutants by Fischer and Sengebusch in 1935 led to NLL genotypes with QA content in grains below 0.05% or even 0.02% (below 500 and 200 mg kg^−1^).^17^, ^24^, ^25^ The sweet lupins carrying this mutation are also referred to as ‘alkaloid‐poor’ lupins.^24^, ^26^ Sweet lupins facilitate grain processing because the reduced QA content eliminates the need for debittering before processing, consumption, or feeding.^19^, ^27^ Nowadays, this mutation is present in almost all NLL varieties on the market.^17^ In Australia and New Zealand, legally binding QA limits of 200 mg kg^−1^ were introduced, whereas in the EU, only a recommended maximum QA content of 200 mg kg^−1^ for food and 500 mg kg^−1^ for feed apply.^21^, ^27^, ^28^, ^29^, ^30^

In addition to the genetic component affecting QA content, converging evidence indicates a stress‐related QA response in sweet NLL, whereby QA content in harvested grains is elevated in stressed plants compared with unstressed counterparts.^25^, ^29^, ^31^, ^32^ Abiotic stressors associated with elevated grain QA content include high temperature, heat during flowering, drought stress before seed filling, potassium deficiency, excessive nitrogen and magnesium availability, soil nutrient imbalances, and low soil pH. These stressors generally increase total QA content in sweet NLL grains by between 5% and 300% compared with unstressed lupins.^16^, ^32^, ^33^, ^34^, ^35^

The QA profile of NLLs is chemically diverse, comprising up to 15 main QAs as well as minor QAs.^30^, ^36^, ^37^ Lupanine is typically the most abundant QA in sweet NLLs and is widely used as the principal marker for total QA content in breeding and toxicological assessments.^16^, ^25^ Alongside lupanine, angustifoline, 13‐hydroxylupanine, and isolupanine are frequently detected at substantial levels, often accounting for the majority of the total QAs.^16^, ^32^ Among these compounds, 13‐hydroxylupanine is the most consistently detected hydroxylated derivative of lupanine.^30^ The QAs exhibiting an abundance at or above 1% of the total QAs are classified as main QAs whereas those below this threshold are considered minor QAs.^36^ At least some of the minor QAs, such as albine, epilupinine, multiflorane, and sparteine are also commonly detected in sweet NLL varieties, although their content typically remains below 1% of the total QAs detected.^34^, ^35^

In addition to overall increases in QA content in response to stress, several studies have reported stress‐induced shifts in the relative abundance of individual QAs, whereby the ratios among QAs change and certain compounds increase disproportionately. Under potassium deficiency, the proportion of 13‐hydroxylupanine increases relative to lupanine.^16^, ^38^ Mechanical wounding represents another stressor that alters QA ratios in sweet varieties, leading to increased relative abundances of angustifoline, isolupanine, and 13‐hydroxylupanine compared with lupanine.^35^ Drought, on the other hand, was shown to decrease the relative amount of lupanine present in the grains.^33^ Overall, the literature shows considerable variability in stress‐induced ratio changes, with only the 13‐hydroxylupanine to lupanine ratio consistently increasing under stress. 13‐Hydroxylupanine can increase significantly with drought, potassium deficiency, and mechanical wounding.^16^, ^33^, ^35^

Numerous studies have documented stress‐induced changes in grain QA content in NLL; however, no study has yet examined the grain QA response of NLL under mixed cropping. The present study addresses this gap by testing whether intercropping NLL with oat alters total grain QA content and the composition of individual QAs. Mixed cropping of pulses with cereals increases biodiversity, enhances system resilience to stress, maintains productivity, and reduces nitrogen fertilizer demand.^39^, ^40^, ^41^ Such systems are thus viable expansions of the suite of tools to adapt to climate change and reduce climate change impact.^42^ A commonly studied field combination is NLL with oat.^43^, ^44^

Agronomically, this pairing combines two established break crops. Narrow‐leaved lupin contributes biological nitrogen fixation and disease resistance benefits in cereal‐dominated rotations, whereas oat is a non‐host or poor host for several key cereal pathogens and is widely recognized as an effective break cereal.^45^ These characteristics facilitate a rapid return to cereals in the subsequent season. Beyond agronomic benefits, the mixture supports high protein production per unit area as pulse–cereal mixtures commonly maintain or increase combined grain and protein yields while reducing nitrogen fertilizer requirements and improving resource use efficiency and system resilience.^7^, ^39^, ^41^ The inherent strengths of NLL, including high seed protein content, strong stress tolerance, atmospheric nitrogen fixation, and relatively low susceptibility to anthracnose make NLL–oat mixtures a strong candidate for sustainable regional protein production under climate change.

A recent study by Andersen *et al*.^46^ observed elevated prenylated flavonoids in NLLs in mixed cropping with barley. Those flavonoids were believed to be connected to a plant's stress response and Andersen *et al*. suggested that mixed cropping with barley is a stressor for the NLLs. Thus, based on the current state of knowledge, it might be expected that mixed cropping with oats would increase the total QA content in NLL grains, with individual QAs increasing to different extents. In particular, it could be expected that the proportion of 13‐hydroxylupanine in comparison with lupanine might increase in mixed cropping. Thus, the aim of this study was to identify, for the first time, the response of the grain QA contents in NLLs in mixed cropping with oats.

## MATERIALS AND METHODS

### Variety selection

Three spring NLL varieties and three spring oat varieties were chosen for co‐cultivation. The NLL varieties were selected based on the QA content accumulated in their grains as reported in breeders’ fact sheets.^31^, ^47^ All chosen NLL varieties were of the branching growth type:

- *Lunabor*, bred by Saatzucht Steinach in Germany – harvested grains were often reported to exceed 500 mg kg^−1^ in total QA;
- *Probor*, bred by Saatzucht Steinach in Germany – harvested grains were reported to contain QAs below and above 500 mg kg^−1^ in total QA;
- *Jowisz* (synonym: Jupiter), bred by HR Smolic in Poland – harvested grains were not reported to exceed 500 mg kg^−1^ in total QA.

Spring oat varieties were selected based on final plant height and early flowering characteristics, using the German Variety Institute list,^47^ to achieve coincident ripening with the NLL varieties:

- *Bison*, bred by Hauptsaaten für die Rheinprovinz GmbH, Germany – a medium tall, early maturing oat variety;
- *Lion*, bred by Nordsaat Saatzucht GmbH, Germany – a medium tall, early maturing oat variety;
- *Troll*, Saatzucht Bauer GmbH & Co. KG, Germany – a short, early maturing oat variety.

### Treatments and experimental design

The NLLs were cultivated in pure stands and mixed stands. In the mixed stands, each NLL variety was co‐cultivated with each oat variety using a replacement design – NLLs were sown at 90% of their pure stand's density, whereas the oats were sown at 10% of their pure stand's density. The low oat proportion was chosen to prevent NLLs from being outcompeted during early growth, given the high competitiveness of oats.^48^, ^49^ Sowing densities for the pure stands were based on local recommendations and adjusted according to seed germination rates (Table 1), which were evaluated in a certified laboratory 2 weeks before sowing, as required by the international seed testing association (ISTA).

**Table 1:** Study treatments: Narrow‐leaved lupin and oat varieties, stand type (pure or mixed), sowing ratios, sowing densities, and seed rates per ha (adjusted to 90% germination). For mixed stands, the first value refers to NLL and the second to oat

Treatment	Stand	Sowing ratios (%)	Sowing density (plants m^−2^)	Amount (kg ha^−1^)
*Lunabor*	Pure	100	120	220
*Probor*	Pure	100	120	170
*Jowisz*	Pure	100	120	214
*Lunabor* + *Bison*	Mixed	90, 10	108/30	198/17
*Lunabor* + *Lion*	Mixed	90, 10	108/30	198/12
*Lunabor* + *Troll*	Mixed	90, 10	108/30	198/11
*Probor* + *Bison*	Mixed	90, 10	108/30	153/17
*Probor* + *Lion*	Mixed	90, 10	108/30	153/12
*Probor* + *Troll*	Mixed	90, 10	108/30	153/11
*Jowisz* + *Bison*	Mixed	90, 10	108/30	193/17
*Jowisz* + *Lion*	Mixed	90, 10	108/30	193/12
*Jowisz* + *Troll*	Mixed	90, 10	108/30	193/11

**Figure 1:**
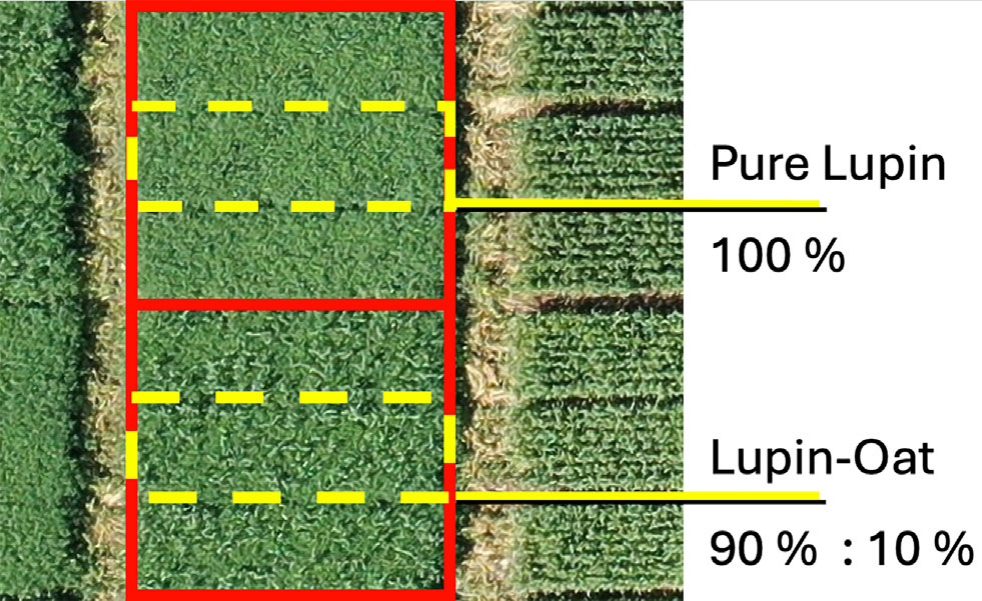
Plot arrangement to minimize border effects. Red lines enclose the three plots of the same treatment, and yellow dashed lines indicate the harvested plots. A 1.2 m mown corridor separated adjacent treatments.

The fields for this 2‐year study were arranged in a randomized complete block design (RCBD), with four blocks. To minimize border effects, each treatment was sown in three adjacent plots, with only the central plot harvested (design shown in Fig. 1). The size of each harvested plot was 7 m^2^.

### Site, husbandry measures and weather data

In both years the sites were located in the same area in Seegräben, Canton of Zurich, Switzerland, and were selected for soil properties suitable for NLL cultivation – a high sand content, absence of free calcium, and pH values ranging from 6 to 6.5. No previous plot‐based trials had been conducted on these fields, resulting in minimal within‐field variability. Table 2 provides an overview of site characteristics and key husbandry dates. Fields were ploughed 3 weeks before sowing and were harrowed 1 week before sowing. Less than 12 h before sowing, the NLL seeds were inoculated with sterile peat inoculum containing the rhizobium *Bradyrhizobium lupinus* (Legume Technology Ltd, Nottingham, UK). The same inoculum batch was used in both years and stored dry and at 10 °C under humidity exclusion. After the inoculation, each 7 m^2^ plot was sown individually in rows spaced 18 cm apart using a plot sowing tractor and real‐time kinematic (RTK)‐GPS orientation. Mechanical weed control, consisting of spring‐tine harrowing and shallow inter‐row cultivation, was performed once after emergence and again during early tillering. Fields were neither fertilized nor treated with pesticides before or during the experiment.

**Table 2:** Field characteristics and husbandry dates of both cultivation seasons

Variable	Site in 2022	Site in 2023
Latitude	47° 20'52.332''N	47° 21'36.252''N
Longitude	8°45'28.836''E	8°45'45.108''E
Pre‐crop	Silage maize	Silage maize
Pre‐pre‐crop	Winter wheat	Winter barley
Sowing	March 10	February 22
Mechanical weeding I	March 17	March 1
Mechanical weeding II	March 31	April 5
Harvest	July 19	July 19
Soil‐type	Sandy loam	Sandy loam
Soil‐pH (H_2_O)	6.2	6.4
Free calcium (HCl 10%)	None	None

Both cultivation seasons were characterized by warm temperatures and high sunshine durations (Table 3). Supporting Information, Figure S1 provides a detailed overview of the weather during the two cultivation seasons.

**Table 3:** Weather data in the cultivation seasons 2022 and 2023, retrieved from the closest official weather station in Seegräben (maximum distance to the site was 4 km)

Parameters	Season 2022	Season 2023	Reference*
Mean temperature (°C)	14.1	13.8	12.3
Precipitation sum (mm)	504	500	530
Mean humidity (%)	69	71	72
Sunshine sum (h)	980	910	802

*The reference provides the averages for the period 1990 to 2020 during the cultivation period. Data were retrieved daily and the average temperature and humidity as well as the sum of precipitation and sunshine hours across the cultivation period were calculated (data retrieved from MeteoSwiss). Further meteorological details are provided in Supporting Information, Fig. S1.

### Harvest and sample preparation

The grains were harvested using an RTK‐GPS‐assisted plot combine harvester. After harvest, the grains were cleaned with a vertical air sifter (in‐house built) and dried to 10% moisture content. In mixed treatments, the oats were separated from the NLLs with a grain cleaner (Westrup, Slagelse, Denmark) with a 6 mm oval‐cut sieve. The cleaned NLL samples were repeatedly divided using a riffle divider (sample splitter RT6.5, Retsch Ltd, Haan, Germany), to obtain representative 150 g NLL samples. These samples were ground using a ZM 200 ultra‐centrifugal mill (Retsch), with a 0.5 mm sieve, and stored in plastic containers at 10 °C.

### Sample extraction and quantification of quinolizidine alkaloids

From NLL grain flour, three representative 2 g samples were suspended in 40 mL of a methanol–water (1:1 v/v) mix, containing 1% (v/v) formic acid. Quantification of QAs in the NLL grains followed the protocol described by Namdar *et al*. 2024.^30^ Linear gradient elution (methanol, %) was applied as follows: 0–1 min – 0%; 8 min – 40%; 12 min – 80%; 12.2–14.2 min – 0%. Liquid chromatography–tandem mass spectrometry was performed on a Waters Acquity ultra‐performance liquid chromatography (UPLC) system coupled to a Xevo TQ‐XS tandem mass spectrometer with an electrospray ionization interface (Waters, Milford, MA, USA). Separation was achieved on an Acquity BEH C18 column (1.7 μm, 100 × 2.1 mm). The limit of quantification (LOQ) was defined as the lowest validated concentration, 0.2 mg kg⁻¹.

Masslynx and Targetlynx software were used to acquire and process the data, respectively. The analysis was repeated three times for each sample. Fourteen QA standards were deployed to analyze the following QAs: albine, anagyrine, angustifoline, *trans*‐13‐cinnamoyloxylupanine, cytisine, epilupinine, 13‐hydroxylupanine, isolupanine, lupanine, lupinine, methylcytisine, multiflorine, sparteine, and thermopsine. Supporting Information, Table S1, provides further details on the standards. The stock solutions were prepared according to the protocol developed by Namdar *et al*. 2024.^30^

### Statistical analysis

All statistical analyses were conducted using R version 4.5.2.^50^ Package management and reproducibility were ensured by using *renv* version 1.0.7 within the R environment.^50^ Principal component analysis and permutational multivariate analysis of variance (PERMANOVA) were performed, and Pearson correlations among the response variables (QAs) were calculated. Bayesian linear mixed models were fitted to the non‐transformed response variables using the R package *blme*.^51^ Each model was run with eight Markov chain Monte Carlo (MCMC) chains, each comprising 3000–4000 iterations, including 1000 warm‐up iterations. This configuration ensured adequate posterior sampling and convergence. Model convergence was assessed using Rhat ≤ 1.01, and sampling efficiency was evaluated using bulk effective sample size (Bulk_ESS_) of 400 or more and tail effective sample size (Tail_ESS_) of 400 or more. Contrast sum parametrization was applied to facilitate interpretation of model coefficients relative to the population mean. Two‐sided posterior probabilities were derived from the credible intervals. Only effects meeting a Bonferroni‐adjusted significance threshold of *P* < 0.05 were considered to be significant.

The main regression model was specified as:

$$\mathrm{QA}∼\text{treatment}+\text{repetition}\times \text{year}+\left(1|\text{block}\_\mathrm{ID}/\text{plot}\_\mathrm{ID}\right)$$

Two ancillary regression models were defined as

$$\mathrm{QA}∼\text{repetition}\times \text{year}+\text{stand}\times \text{lupin}\_\text{variety}+\left(1\;|\;\text{block}\_\mathrm{ID}/\text{plot}\_\mathrm{ID}\right)$$

$$\begin{array}{l} \mathrm{QA}∼\text{repetition}\times \text{year}+\mathrm{oat}\_\text{variety}\times \text{lupin}\_\text{variety}\\ \;+\;\left(1|\text{block}\_\mathrm{ID}/\text{plot}\_\mathrm{ID}\right) \end{array}$$

where *treatment* is the treatment from Table 1, *repetition* is the sample repetition of the QA‐analysis (1–3) and *year* is the cultivation season. The main model evaluated the treatment and year effect. The first ancillary model evaluated the interaction between the NLL variety and its cropping stand. The second ancillary model evaluated the interaction between the NLL variety and the oat variety.

## RESULTS

### Absolute and relative changes in QAs and their total sum in mixed cropping

Among the 14 QAs analyzed, angustifoline, 13‐hydroxylupanine, isolupanine, lupanine, multiflorine, and sparteine were present in all samples, whereas albine was present in 83% and lupinine in 2% of all samples. Lupanine was the most abundant QA, followed by 13‐hydroxylupanine, with contents ranging from 100 to 450 mg kg⁻¹ and 108 to 380 mg kg⁻¹, respectively. The main QAs (abundance ≥ 1% of total QAs),^36^ namely angustifoline, 13‐hydroxylupanine, isolupanine, lupanine, and multiflorine, together accounted for more than 98% of total QAs. The minor QAs, albine and sparteine, occurred at low levels, ranging from below the limit of detection (LOD) to 3 mg kg^−1^. Overall, the NLL variety *Lunabor* showed the highest contents in four out of five QAs, followed by *Probor*. Of all main QAs, except for multiflorine, the lowest content was observed in *Jowisz*.

**Figure 2:**
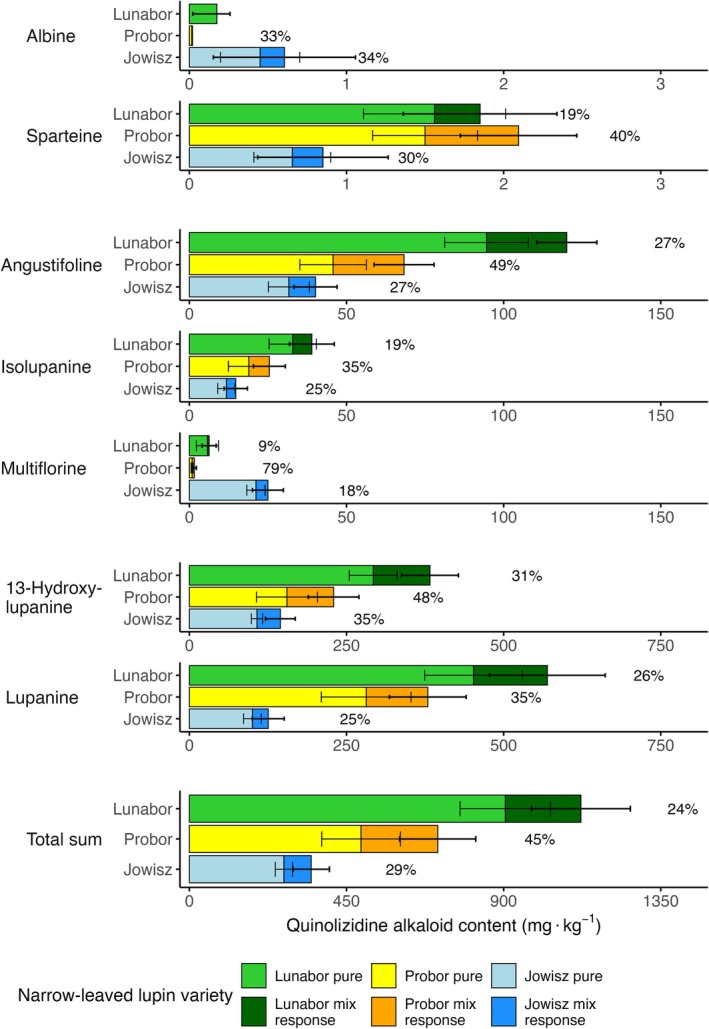
Median responses of all quinolizidine alkaloids in each narrow‐leaved lupin variety averaged across the three oat mixture partners. Data were pooled from both years. Bar height represents median values, with whiskers showing the median absolute deviation in pure and mixed stands. Percentages indicate the median increase in mixture; no percentage is shown if the response was below the limit of quantification (LOQ). Numeric values are provided in Supporting Information, Table S2, and the effect sizes are reported in Table 4.

**Figure 3:**
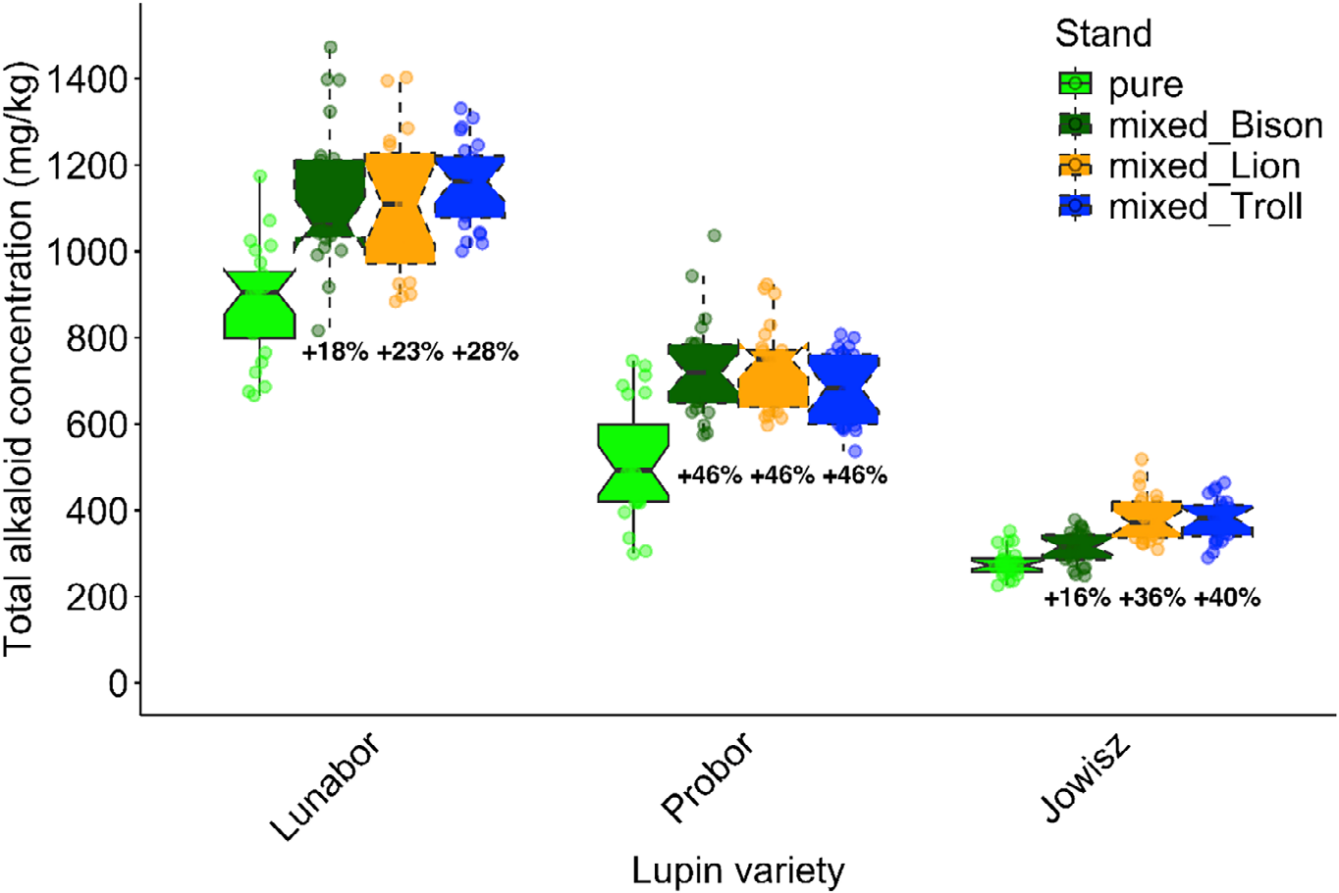
Total quinolizidine alkaloid content in the narrow‐leaved lupin (NLL) varieties and across both stands in mg kg^−1^. The box plots show the total alkaloid content of three lupin varieties (*Lunabor*, *Probor*, and *Jowisz*) cultivated in pure stands and in mixed stands with the three oat varieties. Pure stands are represented in bright green, and mixed stands are depicted in darker shades corresponding to the respective oat variety. The percentage below each boxplot portrays the increase of the quinolizidine alkaloids compared with the lupin's pure stand. Each point represents one measurement. Other than the three mixed treatments of *Probor*, all recorded totals differed significantly.

The outputs of the mixed‐effects models provide an overview of the significant mixed‐cropping effect on QAs in the three NLL varieties (Table 4). Regression results indicate that total QA content increased in mixed stands compared with pure stands. In mixtures with all oat varieties, this increase occurred across all NLL varieties whereas the year had no significant effect on the total grain QA (Fig. 2). *Lunabor* showed the strongest absolute mixed‐cropping response across all mixed treatments with oats (+168 mg kg^−1^, *P* < 0.005), followed by *Probor* (+128 mg kg^−1^, *P* < 0.005) and *Jowisz* (76 mg kg^−1^, *P* < 0.005). The significant oat‐variety interaction (*P* < 0.0001) contributed to variable mixed‐cropping responses; mixtures with *Bison* showed the lowest total grain QA increase, followed by *Troll* and *Lion*. Varietal interactions strongly influenced the mixed‐cropping response, resulting in relative increases ranging from 16% (*Jowisz*–*Bison*) to 46% (all *Probor* mixtures) (Fig. 3). All treatments except the three mixed *Probor* treatments were significantly different from the other treatments (*P* < 0.001). Regardless of cropping stand, *Lunabor* contained the highest QA content, followed by *Probor* and *Jowisz*. Both pure and mixed plots of *Lunabor* consistently exceeded 500 mg kg^−1^ total QA. In *Probor*, the median QA was below 500 mg kg^−1^ in pure stands but rose above that threshold under mixed cropping. By contrast, *Jowisz* never surpassed 500 mg kg^−1^ in either pure or mixed stands.

**Table 4:** Median regression effect size estimates (mg kg^−1^) indicating the change in individual and total QA content. All corrected *p*‐values associated with the effects presented in this table lie below *p* < 0.005. The effect of the repetition indicates the difference between the replicates. Effect rows are only included in the table if they contained significant results. Estimates that were below the limit of quanitifcation (LOQ) or limit of detection (LOD) were indicated as such

Estimated effect size (mg kg^−1^)	Albine	Sparteine	Angustifoline	Isolupanine	Multiflorine	Lupanine	13‐Hydroxylupanine	Total sum
Intercept	0.3	1.3	71	27	10	338	243	**692**
*Lunabor*	>LOQ	+0.2	+40	+14	‐2	+196	+120	**+367**
*Probor*	−0.27	−0.4	−8	+2	−11	+13	−29	**+35**
*Jowisz*	+0.29	−0.6	−32	−12	+13	−209	−91	**−332**
in mixture	<LOQ	<LOQ	+10	+3.2	+1.9	+40	+34	**+91**
*Lunabor* × mixed	<LOD	<LOD	+4	+1.6	<LOD	+24	+13	**+42**
*Probor* × mixed	<LOD	<LOD	−2	<LOQ	<LOD	+5	−2	**−8**
*Jowisz* × mixed	<LOD	<LOD	−6	−1.7	<LOD	−29	−15	**−50**
Year 2022	+0.2	<LOD	+4	+6.5	<LOD	<LOD	<LOD	<**LOD**
Repetition	<LOD	+0.3	−2	+2.5	<LOD	−30	−19	**−62**
*Bison*	<LOD	<LOD	+6	<LOD	<LOD	<LOD	+18	<**LOD**
*Lion*	<LOD	<LOQ	+9.5	+3	<LOD	+43	+29	**+62**
*Troll*	<LOD	<LOD	−2	−4	<LOD	−23	−2	**+43**
*Jowisz* × *Bison*	<LOD	<LOD	−2.5	−0.9	−1	−8.4	−10.5	<**LOD**
*Lunabor* × *Bison*	<LOD	<LOD	<LOD	<LOD	+3.3	<LOD	<LOD	<**LOD**

Across all varieties, angustifoline (+10 mg kg^−1^, *P* < 0.005), 13‐hydroxylupanine (+34 mg kg^−1^, *P* < 0.005) and lupanine (+40 mg kg^−1^, *P* < 0.005) contributed significantly to the total QA mixed‐cropping response. Of all QAs, 13‐hydroxylupanine exhibited the strongest relative increase (*P* < 0.005). Multiflorine, despite being abundantly present in *Jowisz*, exhibited identical absolute increases in all NLL varieties (*P* < 0.005). Minor QAs exhibited similar changes; the sparteine content was elevated in all mixtures, and the albine content also increased through mixed cropping. Only few significant variety interactions between individual QAs were detected.

### Quinolizidine alkaloid profiles

**Figure 4:**
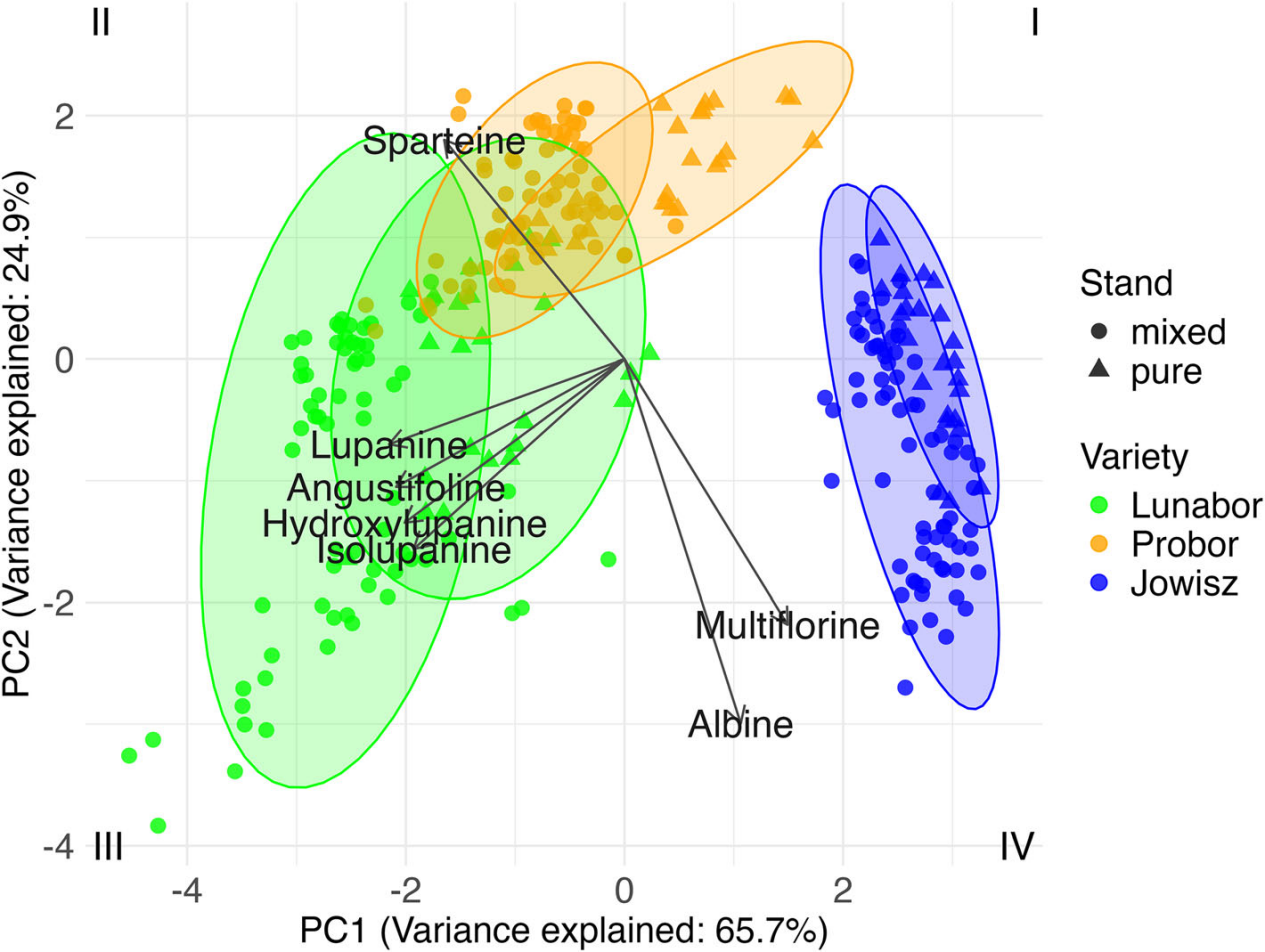
Principal component analysis (PCA) biplot with confidence ellipses and loading vectors, depicting the separation of quinolizidine alkaloid profiles among the three narrow‐leaved lupin varieties and cropping stands. Confidence ellipses (ovals) represent the variability within each variety with a 95% certainty. Each point represents one measurement.

A principal component analysis (PCA) with 95% confidence ellipses showed distinct clustering of *Jowisz*, indicating clear separation of its QA profile from those of *Lunabor* and *Probor* (Fig. 4). The major QAs angustifoline, 13‐hydroxylupanine, isolupanine, and lupanine showed high negative loadings, indicating that principal component (PC) 1 was largely driven by variation in these compounds (PC1 loadings −0.44, −0.42, −0.40, and −0.45, respectively). In contrast, variation along PC2 was mainly associated with the minor QAs albine and multiflorine, which exhibited PC2 loadings of −0.63 and −0.46, respectively, and thus drove separation along this axis. Sparteine showed an opposing contribution to PC2 (loading −0.38), suggesting an inverse relationship with the variation explained by albine and multiflorine. Together, PC1 and PC2 explained 92% of the total variation in the QA profiles. The importance of these loadings was supported by strong and significant correlations among the main QAs driving PC1, with angustifoline, 13‐hydroxylupanine, isolupanine, and lupanine all highly correlated with one another (R^2^ = 0.91–0.97, *P* < 0.001). These compounds were also strongly correlated with the total QA content (R^2^ = 0.93–0.99, *P* < 0.001).

**Figure 5:**
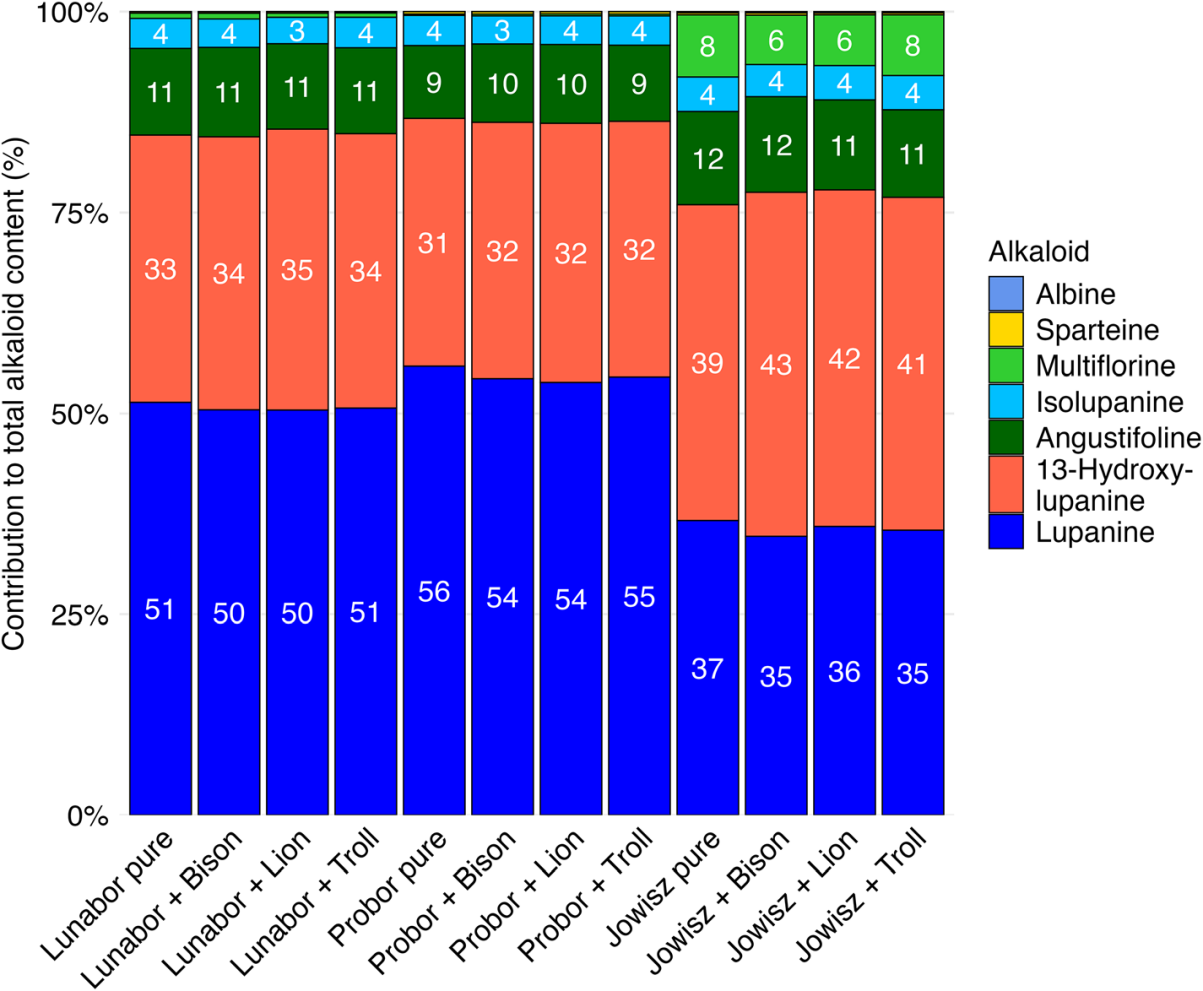
Relative occurrence of individual quinolizidine alkaloids contributing to the total alkaloid content in the three narrow‐leaved lupin (NLL) varieties cultivated in pure and mixed stands. The stacked bar plot illustrates the proportional contributions of the quinolizidine alkaloids to the total content. The numbers printed in the bars represent each alkaloid's relative contribution in percent. Each NLL variety is presented in a pure stand and in mixed stands co‐cultivated with the three oat varieties.

The analysis of the QA profiles across all NLL varieties cultivated in pure stands and mixed stands with the three oat varieties revealed significant variation driven by the NLL variety and the cropping stand (Fig. 5). The PERMANOVA results underlined the different profiles in each variety and between pure and mixed stands (*P* < 0.001), whereas the oat variety companion crop did not show any significant effect. The PERMANOVA results further showed that the NLL variety accounted for 82% of the variance in the QA profiles (R^2^ = 0.82), with a highly significant effect (F = 647, *P* < 0.001). To a lesser but still significant extent, the stand (pure versus mixed) explained 4.3% of the variance (R^2^ = 0.043, F = 12.7, *P* < 0.001). The post hoc pairwise comparisons further highlighted significant differences among all three NLL varieties, revealing distinct QA profiles for *Lunabor* versus *Probor* (R^2^ = 0.56, F = 234, *P* < 0.001), *Lunabor* versus *Jowisz* (R^2^ = 0.87, F = 1212, *P* < 0.001), and *Probor* versus *Jowisz* (R^2^ = 0.77, F = 632, *P* < 0.001). In contrast, the different oat varieties as mixture partners did not significantly interact with the NLL QA profiles.

The coefficient of variation (CV) PERMANOVA analysis of the QA contents across all NLL varieties (*Lunabor*, *Probor*, and *Jowisz*), cropping stand (pure and mixed), and years (2022 and 2023) revealed that the CV did not vary significantly between pure and mixed stands, or years, nor did their interactions.

### Quinolizidine alkaloid ratios

The effects of mixed cropping and oat variety interaction on the ratios of the main QAs (angustifoline, 13‐hydroxylupanine, isolupanine, lupanine, and multiflorine) were evaluated. The only significant mixed‐cropping response was observed in the 13‐hydroxylupanine‐to‐lupanine‐ratio (*P* < 0.005), which increased from 0.57 to 0.62 in mixed stands, representing a 7.4% relative increase in 13‐hydroxylupanine compared with lupanine.

## DISCUSSION

This study identified mixed cropping with oats as a previously unrecognized cause of increased QA accumulation in NLL grains. The extent to which the QA content increased due to mixed cropping is comparable with increases through water stress, increased temperature during flowering, and mechanical damage to leaves.^31^, ^32^, ^33^, ^35^ However, the responses in QA to mixed cropping are at the lower end of documented increases; multiple studies have reported increases ranging from 1.2‐fold up to 11‐fold,^29^, ^37^, ^52^ whereas the increases in the current study remained below 1.5‐fold. The QA increases in response to mixed cropping could be observed across all varietal combinations of NLLs with oats, whereas other studies reported a presence‐absence variation between the investigated NLL varieties, often distinguishing between responding and non‐responding varieties.^32^, ^38^ Other stress responses have also been reported to reduce QA accumulation; for example, Christiansen *et al*.^33^ observed decreased QA levels in NLL grains under drought stress during grain filling. Although year effects on QA content are commonly reported, the mixed‐cropping response in the present study did not differ significantly between years. Overall, within the varieties studied, mixed cropping of NLLs with oats represented a particularly consistent factor leading to increased total grain QA content.

The increase in total QAs under mixed cropping warrants toxicological consideration due to their geno‐ and neurotoxic effects.^19^ Establishing the toxicity of individual QAs for humans and grazing livestock is challenging because dose–response relationships differ substantially between rodents and other mammals or humans.^21^, ^22^ However, the reported toxicities of 13‐hydroxylupanine and lupanine are identical in rats and similar in mice.^21^, ^22^ Given the comparable QA profiles in the present study, it is reasonable to assume that the increase in total grain QA is directly proportional to an increase in grain toxicity for food and feed. Consequently, mixed cropping of NLLs with oats elevates the toxicity of NLL grains and must be considered in cultivation for these purposes. Careful selection of both NLL and oat varieties, with attention to their interactions, is therefore essential.

In the present study, variations in total grain QAs were mostly attributed to NLL varietal differences and their interactions with the oat varieties, with resource competition and oat allelopathy possibly playing a crucial role. The NLLs and oats in mixed cropping compete for soil resources, and NLLs were shown to accumulate grain QAs in a variety‐specific manner in responses to differences in nitrogen, phosphorus, potassium, and drought conditions.^31^, ^32^, ^38^ Frick *et al*.^32^ demonstrated that NLL varieties can exhibit distinct responses to single stressors, multiple stressors, or their interactions. Oats were also shown to display genotype‐specific allelopathic effects, suppressing the growth of neighboring plants and thereby inducing stress;^52^, ^53^ for example, oat root exudates reduced growth of *Sinapis arvensis*.^52^ The combination of soil resource competition, genotype‐specific oat allelopathy, and variety‐specific NLL stress responses thus accounts for the observed differences in grain QA accumulation in this study.

The increase in the main individual QAs in mixed cropping was proportional to the total increase, resulting in identical QA profiles in pure and mixed stands. This consistency was further supported by the uniform ratios of the main QAs (except for 13‐hydroxylupanine to lupanine), their strong positive correlations, and the absence of significant differences in their coefficients of variation. Such a uniform increase across all main QAs and NLL varieties is unusual; several studies evaluating individual QA responses to various stressors reported a more heterogeneous pattern, with increases differing within and between NLL varieties and across stress types, and relative changes varying by up to two orders of magnitude.^33^, ^35^, ^37^ Interestingly, the evidence points not only to a near‐absence of QA biosynthesis within NLL grains, but also to the fact that the alkaloids detected in the seeds were translocated there from the foliage.^54^, ^55^ Thus, unlike other stressors, mixed cropping probably leads to an overall up‐regulation of the main QA biosynthesis or transport into the grains. Shade‐induced nitrogen redistribution is an unlikely driver of the observed rise in QA levels: under low light, plants shift nitrogen away from mobile nitrogen‐rich metabolites such as QAs, and towards nitrogen‐rich photosynthetic proteins.^56^ A tissue‐based metabolomics approach could clarify whether mixed cropping with oats enhances QA synthesis or promotes translocation of QAs into the grains.

Quinolizidine alkaloids are not known to impact other plants; therefore, the increase of QAs in the NLL grains is unlikely to increase their competitiveness against oats. Thus, a tangential (indirect) mechanism is a more probable way of action: mixed cropping is a stressor for NLL. Such a tangential stress response has also been proposed by Czepiel *et al*.^57^ to explain QA increases following anthracnose infection. Although QAs had no direct role in defending against anthracnose, infection led to elevated QA levels in NLLs.^57^ The QA increase in NLL grains under mixed cropping supports the interpretation that oats act as a stressor. This conclusion is reinforced by the observed shift in the 13‐hydroxylupanine‐to‐lupanine ratio, which has been reported to increase under various stress conditions.^16^, ^33^, ^35^ Based on these results, it can be proposed that mixed cropping with oats imposes stress on NLL, triggering an up‐regulation of grain QA accumulation.

## CONCLUSION

The increased QA content and, consequently, the elevated toxicity of NLL grains under mixed cropping with oats may limit their suitability for food and feed, with potential negative implications for feedlots, processors, and consumers. Total grain QA content and the response to mixed cropping varied up to threefold among different NLL–oat variety combinations, highlighting the importance of variety selection as an immediate mitigation strategy.

To maintain the added value of NLL–oat mixed cropping systems, two approaches are proposed. First, the genetic basis underlying QA increases under stress should be identified to enable breeding of stress‐resistant NLL varieties that do not respond with elevated QA levels. It is also important to determine whether the effects of multiple stressors on grain QA accumulation are additive or synergistic, and whether oats could be replaced by a companion cereal that induces less stress or lower QA increases.

Second, given the protective role of QAs in plant defense, the concept of ‘bitter/sweet’ genotypes, as first proposed by Wink *et al*.,^58^ offers a promising strategy. In these genotypes, vegetative tissues contain elevated QAs (‘bitter’), whereas grains remain low in QAs (‘sweet’). As QAs are not synthesized *de novo* in NLL grains but are translocated from other tissues,^54^, ^55^, ^58^, ^59^ targeted breeding could optimize this ‘bitter/sweet’ pattern. This approach would mitigate grain toxicity while preserving QAs’ defense functions, enabling the cultivation of stress‐resistant or ‘bitter/sweet’ genotypes. Such varieties could enhance the broader adoption of NLLs as a climate‐resilient pulse crop, providing added value for producers, the food industry, and consumers.

## FUNDING INFORMATION

This project was carried out within the Horizon 2020‐funded European project CROPDIVA (ID: 101000847). The authors gratefully acknowledge the financial support that made this study possible.

## CONFLICT OF INTEREST

The authors declare no conflict of interest.

## Supporting information


**Figure S1:** Precipitation and daily average temperatures of the months March to July for the years 2022 and 2023. The bars represent the precipitation in mm, the dotted line the average temperature in degrees Celsius.

**Table S1:** Overview over the quinolizidine alkaloid standards used. The table provides information on their chemical forms, suppliers, article codes, CAS numbers and the purities.

**Table S2:** Median values and their median absolute deviation of all measured quinolizidine alkaloids in mg kg^−1^ across both cultivation seasons provided for each investigated narrow‐leaved lupin variety in pure stand and mixed stand (in mixed cropping with oats). All contents are based on lupin dry‐weight. The mixed stand is a summary of the three mixed treatments of each narrow‐leaved lupin variety and the three oat varieties. All values for epilupinine and lupinine were zero and are thus not shown.

## Data Availability

The data that support the findings of this study are available from the corresponding author upon reasonable request.
