# Supplementary material for: Elevated quinolizidine alkaloid content in grains of sweet narrow‐leaved lupins when intercropped with oats
Source: J Sci Food Agric. 2026 Feb 11;106(5):2917–27. doi: 10.1002/jsfa.70396 (PMC12967730; doi:10.1002/jsfa.70396)
Supplement: Supplementary file 1 — Figure S1: Precipitation and daily average temperatures of the months March to July for the years 2022 and 2023. The bars represent the precipitation in mm, the dotted line the average temperature in degrees Celsius. [file JSFA-106-2917-s002.pdf]

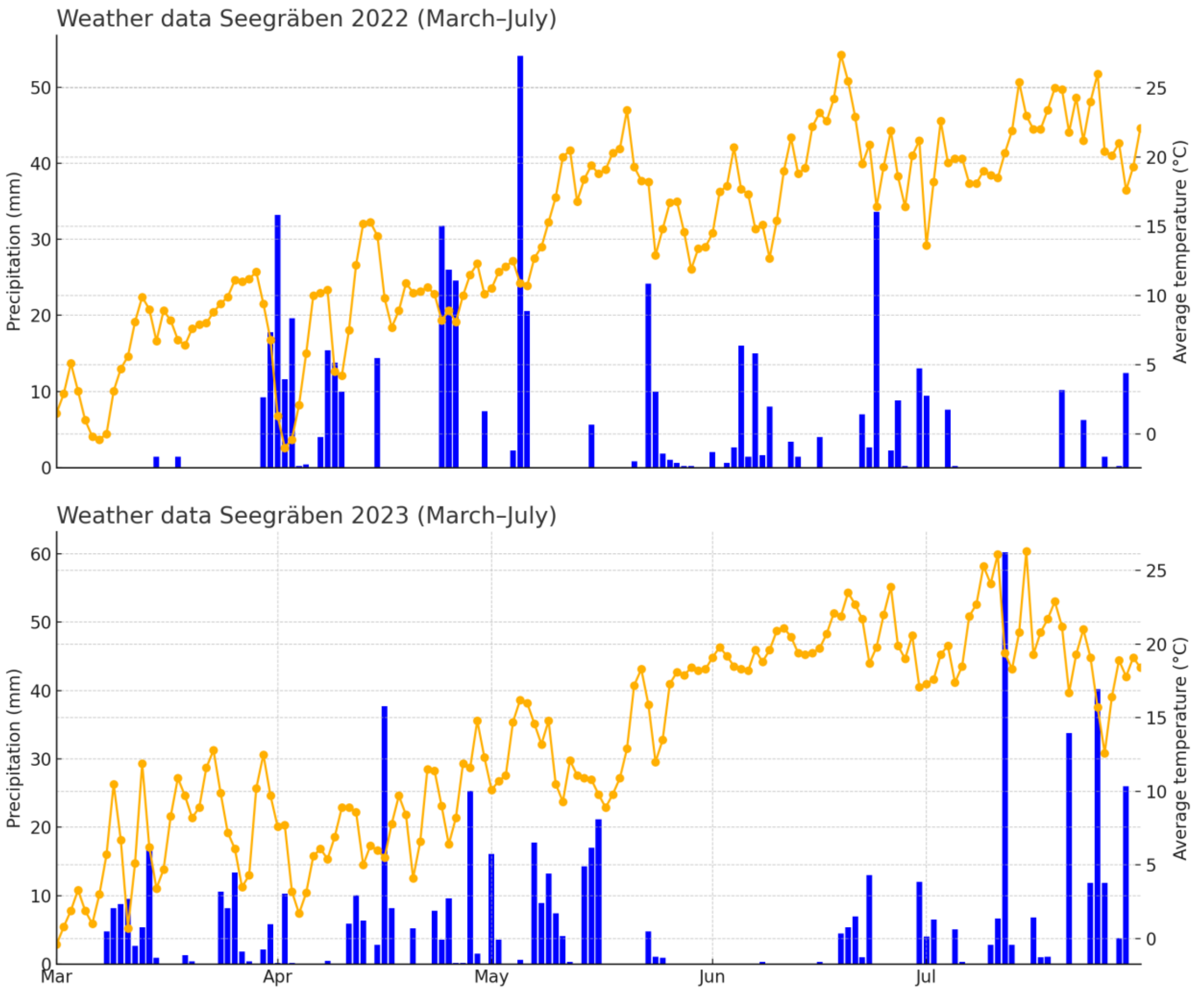

Figure S1: *Precipitation and daily average temperatures of the months March to July for the years 2022 and 2023 in Seegräben, Switzerland. The bars represent the sum of precipitation in mm, the dotted line the average daily temperature in degrees Celsius.*
