# Supplementary material for: Elevated quinolizidine alkaloid content in grains of sweet narrow‐leaved lupins when intercropped with oats
Source: J Sci Food Agric. 2026 Feb 11;106(5):2917–27. doi: 10.1002/jsfa.70396 (PMC12967730; doi:10.1002/jsfa.70396)
Supplement: Supplementary file 2 — Table S1: Overview over the quinolizidine alkaloid standards used. The table provides information on their chemical forms, suppliers, article codes, CAS numbers and the purities. [file JSFA-106-2917-s003.pdf]

Table S1: Overview over the quinolizidine alkaloid standards used. The table provides information on their chemical forms, suppliers, article codes, CAS numbers and the purities.

Quinolizidine alkaloid	Supplier	Article code	CAS number	Purity (%)
Albine, as hydrochloride	PhytoLab	86291	53915-26-7	100
Anagyrine, as hydrochloride	PhytoLab	83211	74195-83-8	99.4
Angustifoline	PhytoPlan, Germany	6320-95	550-43-6	99.2
Trans-13α-Cinnamoyloxylupanine	Biosynth	FC65854	5835-04-1	> 98
Cytisine	PhytoLab	83219	485-35-8	99.9
Epilupinine, as hydrochloride	PhytoPlan, Germany	Test standard	486-71-5	–
13(-α)-Hydroxylupanine	PhytoPlan, Germany	6321-95	1148-95-6	97.6
D-α-Isolupanine, as perchlorate	LGC	TRC-1820900	14691-01-1	98
D-Lupanine, as hydrochloride	PhytoPlan, Germany	85755	1025-39-4	96.2
(-)-Lupinine	Extrasynthese, France	0694	486-70-4	95
Methylcytisine	PhytoLab, Germany	83219	486-85-1	99
Multiflorine	PhytoPlan, Germany	6324-95	529-80-6	> 99.5
(-)-Sparteine	Sigma-Aldrich, Netherlands	900264	90-39-1	98
