# Supplementary material for: Elevated quinolizidine alkaloid content in grains of sweet narrow‐leaved lupins when intercropped with oats
Source: J Sci Food Agric. 2026 Feb 11;106(5):2917–27. doi: 10.1002/jsfa.70396 (PMC12967730; doi:10.1002/jsfa.70396)
Supplement: Supplementary file 3 — Table S2: Median values and their median absolute deviation of all measured quinolizidine alkaloids in mg kg−1 across both cultivation seasons provided for each investigated narrow‐leaved lupin variety in pure stand and mixed stand (in mixed cropping with oats). All contents are based on lupin dry‐weight. The mixed stand is a summary of the three mixed treatments of each narrow‐leaved lupin variety and the three oat varieties. All values for epilupinine and lupinine were zero and are thus not shown. [file JSFA-106-2917-s001.pdf]

**Table S2:** Median values and their median absolute deviation of all measured quinolizidine alkaloids in mg kg$^{-1}$ across both cultivation seasons provided for each investigated narrow-leaved lupin variety in pure stand and mixed stand (in mixed cropping with oats). All contents are based on lupin dry weight. The mixed stand is a summary of the three mixed treatments of each narrow-leaved lupin variety and the three oat varieties. All values for epilupinine and lupinine were zero and are thus not shown.

| Quinolizidine alkaloid (mg kg$^{-1}$) | Lunabor pure | Lunabor mixed | Probor pure | Probor mixed | Jowisz pure | Jowisz mixed |
|---|---|---|---|---|---|---|
| Albine | 0.2 ± 0.1 | 0.1 ± 0.3 | 0.0 | 0.0 | 0.4 ± 0.2 | 0.6 ± 0.3 |
| Sparteine | 1.6 ± 0.3 | 1.8 ± 0.4 | 1.5 ± 0.2 | 2.1 ± 0.3 | 0.7 ± 0.3 | 0.8 ± 0.3 |
| Angustifoline | 95 ± 12 | 120 ± 12 | 46 ± 10 | 68 ± 9 | 32 ± 5 | 40 ± 6 |
| Isolupanine | 33 ± 6 | 39 ± 9 | 19 ± 6 | 25 ± 5 | 12 ± 2 | 15 ± 3 |
| Multiflorine | 5.7 ± 2.7 | 6.3 ± 3.5 | 0.9 ± 0.4 | 1.6 ± 0.4 | 21 ± 3 | 25 ± 4 |
| 13-Hydroxylupanine | 292 ± 32 | 383 ± 40 | 155 ± 31 | 229 ± 30 | 108 ± 11 | 145 ± 20 |
| Lupanine | 452 ± 58 | 570 ± 83 | 281 ± 64 | 379 ± 44 | 100 ± 12 | 125 ± 20 |
| **Total sum** | **905 ± 102** | **1122 ± 133** | **492 ± 108** | **712 ± 82** | **273 ± 24** | **350 ± 45** |
